# Lectures on Natural and Difficult Parturition

**Published:** 1848-01

**Authors:** 


*Lectures on Natural and difficult Parturition.* By Edward William Murphy, A. M., M. D. *Prof. of Midwifery, University College, London, &c. &c. New York, S. S. & W. Wood,* 1846.

The above work has been sometime before the American public, and an apology may be due the enterprising publishers for permitting it to remain so long unnoticed in our columns.

The well known reputation of Prof. Murphy, the high position which he holds, furnish a guarantee for the ability of the work, and, yet, at this time, when the press teems with systematic works on obstetrics, emanating from the highest authorities, combined with the well known predilections for systematic works in preference to monographic, we fear the publishers may not find the publication a very profitable one. The truth is, that in this conntry the purchasers of medical books are students, and young physicians entering on practice; and, as a matter of course, they purchase the systematic works used as text books in the schools.

Doct. Murphy, in the preface, informs us that the lectures "are the substance of those delivered at the University College on the subject of natural and difficult Parturition; he adds, "the order of the course has been slightly altered by the introduction of the first two lectures on the obstetric anatomy of the pelvis."

The two first lectures contains a minute and accurate description of the obstetric anatomy of the Pelvis, but nothing worthy of notice.

The third lecture gives his definition of labour, which is "*the action of the uterus to expel its contents when the fœtus is sufficiently mature to sustain respiratory life.*"

After considering the classifications adopted by different writers; he makes the following division of labours: "natural, difficult, preternatural, and complex." In his division of the different stages of labours, he prefers that of Denman, the 1st stage being the dilitation of the os uteri; the 2d the expulsion of the child; the 3d, the separation of the placenta. The work is illustrated by plates, which are well executed, representing the different layers of muscular fibres of the uterus. The author dwells at considerable length on the action of the several layers in the process of parturation. We were not a little surprised to find so accurate and intelligent a writer assuming the position that there is no evidence of the neck of the uterus containing muscular fibres. He says: "it seems more probable that the highly condensed tissue that form the cervix of the virgin uterus, still retains in its altered state, many of the original characters; that this tissue, although more unfolded, is still sufficiently compact, and elastic, to offer a great degree of resistance, and that its dilitation is effected by the incessantly repeated efforts of the uterus, slowly overcoming and expanding it." We conceive it is only necessary to observe closely the phenomena of a single labour, to satisfy the most obtuse how perfectly are facts at variance with the above description; instead of the slow gradual yielding of a firm dense substance to an impelling force, we find the mouth of the uterus contracting firmly with each contraction of the uterus, relaxing again as this organ is relaxed, and contracting as it contracts, yielding a little each time, until it is fully open. The prompt and marked effect of blood letting in producing relaxation of a rigid os uteri, is well known to every practitioner. No such effect could be produced if Doctor Murphy were correct. We have been long satisfied that the account given by Denman, and most subsequent writers, as to the manner in which a premature rupture of the membranes of the uterus renders labour tedious is erroneous, and we believe it has often led to bad practice. We will suppose, for instance, that an os uteri is dilated to the size of a half dollar, the membranes are ruptured, and the waters discharged, the uterus now contracts down about the child, by what Dewees terms the tonic contraction, the cervix partakes of this contraction, and, on examination, the os uteri is found reduced to the size of a two-shilling piece, and the process of dilitation has again to be repeated.

It is true that muscular fibers are not as abundant, or as fully developed as in the fundus and body, and probably, the longitudinal fibres of the body, with the exception of a few small fasiculi, cannot be traced into the cervix, but that muscular fibres exist, we consider as evident as that they exist in any part of the uterus.

Doct, M. dissents from the description given by "Wigerent," and adopted by "Rigby," that the contractions are from the cervix to the fundus. He thinks the reverse is the case, and that the contractions begin at the fundus. The observations of the author goes to confirm the accurate description of Naegele in his account of the manner in which the head passes through the pelvis, and that the 3d and 4th positions of Baudeloque, were most frequently in the progress of the labour changed into the 2d and 1st.

Lectures V and VI give a concise, and judicious description of the management of "natural labour." The VII lecture is on the subject of "difficult labours." The causes of difficult labour are divided into insufficient action of the uterus, and rigidity of the passages. In treating ot the subject of rigid os uteri, Doct. M. very properly directs that we should refrain from all attempts at dilitation, but omits to mention the practice recommended by the late Prof. Dewees, viz: blood letting, which has almost a magical effect in producing relaxation.

Lectures VIII, IX and X, are on laborious labour, and contains nothing new. Prof. M. has the common prejudice of English writers against the use of the forceps, unless the head is resting at the inferior strait, and is decidedly opposed to their use in cases of imperfect or locked-head. Lecture XIII on obstetric instruments. The work closes with a summary of the principal rules laid down in the lecture, arranged in the form of aphorisms, to which are added some of the aphorisms of Denman. Notwithstanding the few strictures which we have advanced on some of the opinions of the author, we would commend the work to the profession, as containing an able summary of British practice and doctrine on the subject of natural and difficult parturition.

Dec. 1st 1847. C. B. C.

